# Generation of a Polyclonal Antibody against the Mouse Metal Transporter ZIP8

**DOI:** 10.3390/antib10020016

**Published:** 2021-04-21

**Authors:** Guojun Wei, Yuze Wu, Ningning Zhao

**Affiliations:** Department of Nutritional Sciences, The University of Arizona, Tucson, AZ 85721, USA; gwei@email.arizona.edu (G.W.); yuzewu@email.arizona.edu (Y.W.)

**Keywords:** ZIP8, manganese, antibody

## Abstract

ZIP8 is a newly identified metal transporter. In human patients, mutations in *ZIP8* result in severe manganese deficiency, suggesting a critical role for ZIP8 in regulating systemic manganese homeostasis. In mice, the deletion of ZIP8 recapitulates the symptoms of patients with *ZIP8* mutations. However, further studies using mouse models to examine ZIP8′s function were hindered by the lack of suitable antibodies to detect endogenous ZIP8 protein. In this study, we report the design, generation, and validation of a polyclonal antibody against mouse ZIP8. We have demonstrated that the newly generated antibody can be reliably used in immunoblotting analysis to detect endogenous ZIP8 protein in mouse tissues. The successful generation and validation of anti-mouse ZIP8 antibody provide opportunities to further examine the function and regulation of this metal transporter. In addition, our study may provide valuable insights into the future development of antibodies targeting polytopic membrane proteins.

## 1. Introduction

Antibodies serve as critical tools in a variety of biological research, including the examination of nutrient metabolism. As an essential nutrient, manganese is required for different cellular processes, including gluconeogenesis, protein glycosylation, urea formation, and mitochondrial antioxidant defense [1,2,3]. To carry out these fundamental functions, manganese needs to be imported into the cells by metal transporters. ZIP8 is a polytopic membrane protein that facilitates manganese accumulation in cells [4,5,6]. In humans, mutations in *ZIP8* cause severe manganese deficiency, indicating that ZIP8 plays an important role in regulating physiological processes relevant to systemic manganese homeostasis. However, the precise function of ZIP8 in manganese metabolism remains to be determined.

Similarly to patients carrying *ZIP8* mutations, *Zip8* knockout (*Zip8*^-/-^) mice developed manganese deficiency, suggesting that mice could serve as an animal model to further study ZIP8′s function. To study the function and regulation of proteins, immunoblotting analysis is commonly used for the measurement of protein expression. This assay requires a probe antibody that can recognize the protein of interest. Due to the lack of reliable antibodies that could effectively detect endogenous mouse ZIP8 (mZIP8) in immunoblotting assays, prior studies mainly used epitope-tagged variants of ZIP8 and ectopic expression systems to examine the levels of protein expression [4,6,7]. The knowledge about the expression and regulation of endogenous mZIP8 is limited. Therefore, it is of critical need to develop antibodies that can reliably detect mZIP8 in mouse tissues, to facilitate further investigations of the function and regulation of this metal transporter.

To generate antibodies, one frequently used approach is to produce a short peptide of 10 to 20 amino acids that is predicted to represent a region of the targeted protein [8,9,10]. The peptide needs to be further conjugated to a carrier protein, such as the keyhole limpet hemocyanin (KLH), to increase the immunogenicity. After administration into animals, the host immune system will react to the peptide–KLH conjugate to generate antibodies against the target protein. One drawback of this short-peptide-based approach is that many anti-peptide antibodies can react with the peptide, but fail to recognize the protein from which the peptide sequence was selected, especially for multi-transmembrane proteins [11,12]. Another approach for antibody generation is to produce the full-length target protein as the immunogen for inoculation of animals. However, due to low levels of protein expression and the tendency to form aggregates [13,14,15,16], it is very difficult to express the full-length eukaryotic multi-transmembrane proteins in *E. Coli*, which is the most popular host system used for the production of membrane proteins [17,18,19].

We aimed to employ an alternative approach to generate the anti-mZIP8 antibody by using a glutathione S-transferase (GST) fusion protein with fragment covering about 100 amino acids of the target protein’s unique sequence. This approach has been widely used to generate polyclonal antibodies [20,21]. In this study, we report the design, production, and validation of the antibody for mZIP8. The successful generation and validation of this antibody should provide opportunities to reveal new information concerning the protein expression profiles of this transporter in mice and to further investigate its function in nutrient metabolism. Moreover, our study may provide valuable insights for future developments of antibodies for polytopic membrane proteins.

## 2. Materials and Methods

### 2.1. Bioinformatic Analysis

Amino acid sequences were aligned using Vector NTI 11.0 (Invitrogen, Carlsbad, CA, USA). Prediction of transmembrane domains and topology of proteins was performed using the online servers of HMMTOP [22], MEMSAT [23], TOPCONS [24], and SPOCTOPUS [25].

### 2.2. Plasmid Construction

The sequences encoding serine 30 to serine 124 of mZIP8 were amplified by polymerase chain reaction (PCR) using pCMV-Entry-mZIP8 expression vector (Origene, Rockville, MD, USA) as the template. The following primer sets were used for amplification: mZIP8 forward 5′-ATA AA GGA TCC AAA GTG AGG ATG TGC TGA GCG T-3′; reverse 5′-AAT ATG AAT TCT CAA CTG GGC TTT GCG TTG TGC-3′. To assist the cloning process, BamHI and EcoRI sites were added to the forward and reverse primers, respectively. The amplification products were loaded onto a 2% agarose gel for electrophoresis. The specific bands were excised from the gel, purified using Wizard SV Gel and PCR Clean-Up system (Promega, Fitchburg, WI, USA), digested with BamHI and EcoRI, and subsequently cloned into pGEX-3X vector (Addgene, Watertown, MA, USA) to produce the bacterial expression plasmid encoding mZIP8-GST fusion protein.

### 2.3. Expression and Purification of GST Fusion Proteins

To express the recombinant protein, the pGEX-3X plasmid encoding GST or mZIP8-GST fusion protein was transformed into Rosetta-2 strains of *E. Coli*. The antibiotic-resistant colony was selected and cultured at 37 °C. An aliquot of 500 μL overnight culture was diluted into 4 L of Luria–Bertani (LB) medium and cultured at 37 °C with shaking at 200 rpm. When the bacteria culture reached a density of 0.6 (OD_600_), isopropyl-β-D-thiogalactoside (IPTG) was added to a final concentration of 1 mM to induce the expression of recombinant proteins. After 6 h of induction, the culture was centrifuged at 3500 rpm at 4 °C and the supernatant was discarded. The bacterial pellet was re-suspended in ice-cold cell lysis buffer (50 mM NaCl, 50 mM Tris, 5 mM EDTA, 1× protease inhibitor, pH 7.3) and lysed using a French press. The cell lysates were centrifuged in a JA-17 rotor at 11,000 rpm for 1 h at 4 °C to clear the cell debris, and then applied to the glutathione sepharose to allow the binding of GST or GST fusion protein. The elution buffer (0.1 M glycine-HCl, pH 2.5) was added to elute the bound proteins. Subsequently, proteins were concentrated using Amicon Ultra-4 centrifugal filters with a 10 kDa cut-off (Millipore, Burlington, MA, USA).

### 2.4. Affinity Purification of Antibodies

The immunization procedure was performed by the Pocono Rabbit Farm and Laboratory (Canadensis, PA, USA). The antisera were used to purify antigen-specific antibodies. To cross link GST and GST fusion proteins to the glutathione sepharose, a column with 2 mL sepharose (Cytiva, Marlborough, MA, USA) was equilibrated with 5 mL binding buffer (140 mM NaCl, 2.7 mM KCl, 10 mM Na_2_HPO_4_, 1.8 mM KH_2_PO_4_, pH 7.3). Pre-cleared bacterial lysates containing either GST or GST fusion proteins were incubated with the sepharose for 1 h at 4 °C. The column was then washed 3 times with binding buffer and allowed to drain completely after the last wash. To purify antibodies, 2 mL of anti-serum was incubated with GST-linked column that was pre-washed with 20 mL TBS (10 mM Tris/HCl, 150 mM NaCl, pH 7.50). After 1 h of incubation at 4 °C, the column was eluted by gravity flow and washed with 5 mL TBS. The eluate was then incubated with GST fusion protein-linked column for 1 h at 4 °C. The column was washed with 20 mL TBS and then 10 mL 0.1× TBS. Antibodies were eluted with 0.8 mL elution buffer into 1.5 mL tubes pre-loaded with 0.2 mL neutralization solution (50 mM Tris-HCl, pH 8.0). A total of 10 eluate fractions were collected for further analysis.

### 2.5. Cell Culture and Transfection

HEK 293 cells were grown in Dulbecco’s Modified Eagle’s Medium (DMEM, Sigma-Aldrich, St. Louis, MO, USA) with 4.5 g/L glucose, 4 mM L-glutamine, 1 mM sodium pyruvate, and 10% fetal bovine serum (FBS, VWR, Radnor, PA, USA), and maintained in an incubator with 5% CO_2_ at 37 °C. Plasmids encoding FLAG-tagged mZIP8 (pCMV-Entry-hZIP8) was purchased from Origene. For transient transfection, cells were seeded at 40% confluency in 6-well culture plates. Transfection began 24 h after seeding with 0.4 µg of plasmid DNA, 3.2 µL of enhancer, and 10 µL of Effectene reagent (Qiagen, Germantown, MD, USA). Transfection was carried out for 48 h before further analysis. To knock down the expression of FLAG-tagged mZIP8 protein, 24 h after the plasmid transfection. siRNA targeting mouse ZIP8 (5′-GCG AGG AUC UAA GAA AGC ACA ACG C-3′) was transfected using the Lipofectamine RNAiMAX transfection reagent (Thermo Fisher Scientific, Waltham, MA, USA). A scramble siRNA was used as the negative control. The siRNA transfection procedure was performed per the manufacturer’s protocol using 30 pmol of siRNA and 9 µL of transfection reagent. Cell lysates were collected 24 h after the siRNA transfection.

### 2.6. Animals and Genotyping

Procedures for animal experiments were approved by the Institutional Animal Care and Use Committees (IACUC) of the University of Arizona. Animal cages containing less than 5 mice were kept at 21–22 °C with 12 h light/dark cycles. Mice were provided with tap water ad libitum and fed the NIH-31 irradiated traditional rodent diet (Teklad 7913; Envigo, Indianapolis, IN, USA). *Zip14* knockout (*Zip14*^-/-^) mice were purchased from Mutant Mouse Resource and Research Centers, USA. Conventional whole-body knockout of *Zip8* is embryonic lethal in mice [26]. To generate inducible *Zip8* knockout (*Zip8*^-/-^), mice carrying *Zip8* conditional allele (*Zip8*^flox/flox^) (purchased from Taconic, Germantown, NY, USA) were cross bred with mice expressing Cre-ERT2 (tamoxifen-inducible estrogen receptor) fusion protein under the control of the ubiquitin promoter (Ubc-Cre) (obtained from the Jackson Laboratory, Bar Harbor, ME, USA). *Zip8*^flox/flox, Ubc-Cre^ mice were injected with tamoxifen dissolved in corn oil (100 mg/kg body weight) for 5 consecutive days to inactivate *Zip8*. Mouse Direct PCR kit (Bimake, Houston, TX, USA) and the following primers were used to determine mouse genotypes. For *Zip14*^-/-^ mice: DNA506-100, 5′-TCA TGG ACC GCT ATG GAA AG-3′; DNA506-101, 5′-GTG TCC AGC GGT ATC AAC AGA GAG-3′; Neo3a, 5′-GCA GCG CAT CGC CTT CTA TC-3′; DNA506-6, 5′-TGC CTG GCA CAT AGA ATG C-3′. For *Zip8*^flox/flox^ mice: 2476_27, 5′-CAG GGT TTC TCT GTG TAA CAG G-3′; 2474_28, 5′-AGT GTA CAG GCT CCA GCT ACC-3′. For Ubc-Cre mice: 25285, 5′-GAC GTC ACC CGT TCT GTT G-3′; oIMR7338, 5′-CTA GGC CAC AGA ATT GAA AGA TCT-3′; oIMR7339, 5′-GTA GGT GGA AAT TCT AGC ATC ATC C-3′; oIMR9074, 5′-AGG CAA ATT TTG GTG TAC GG-3′. All mice were sacrificed after anaesthetizing with ketamine/xylazine. Mouse tissues were immediately frozen in liquid nitrogen and stored at −80 °C for further analyses.

### 2.7. Sodium Dodecyl Sulfate Polyacrylamide Gel Electrophoresis (SDS-PAGE) and Immunoblotting

HEK293 cells or mouse tissues were lysed in NETT buffer (150 mM NaCl, 5 mM EDTA, 10 mM Tris, 1% Triton X-100, 1× Protease inhibitor cocktail). Protein concentrations of the lysates were determined by the RC DC assay (Bio-Rad Life Science, Hercules, CA, USA). Tissue lysates with equal amounts of proteins (40–60 µg/sample) were mixed with 1× Laemmli buffer and incubated at 37 °C for 30 min. Proteins were electrophoretically separated on SDS/10% polyacrylamide gels and transferred to nitrocellulose membranes (GVS, Sanford, ME, USA). After 1 h blockage of non-specific binding sites with blocking buffer (5% non-fat dry milk in TBST (10 mM Tris/HCl, 150 mM NaCl, 0.1% 1mL Tween-20, pH 7.5)) at room temperature (RT), membranes were incubated with rabbit anti-mZIP8 or anti-mZIP14 antibody (1:1000) overnight at 4 °C. Membranes were washed 4 times with TBST (5 min/each) and incubated for 1 h at RT with donkey anti-rabbit horseradish peroxidase (HRP)-conjugated secondary antibody (1:5000) (GE healthcare, Chicago, IL, USA). Blots were washed twice (5 min each) with TBST, followed by two washes in TBS prior to imaging. In addition, since the pCMV-Entry vector contained a FLAG epitope, a mouse monoclonal HRP-conjugated anti-FLAG antibody (1:6000) (Sigma-Aldrich) was used to detect FLAG-tagged mZIP8 overexpressed in HEK293 cells. Blots were developed using enhanced chemiluminescence (SuperSignal West Pico, Thermo Fisher Scientific) and the ChemiDoc MP Imaging System (Bio-Rad Life Science). To confirm equivalent loading, blots were stripped for 15 min in Restore PLUS Western Blot Stripping Buffer (Thermo Fisher Scientific), washed 4 times in TBS (5 min each), blocked for 1 h in blocking buffer, and reprobed with HRP-conjugated anti-GAPDH (1:10,000) antibodies (Proteintech, Rosemont, IL, USA).

## 3. Results

### 3.1. Topological Analysis and Signal Peptide Prediction of mZIP8

**Figure 1:**
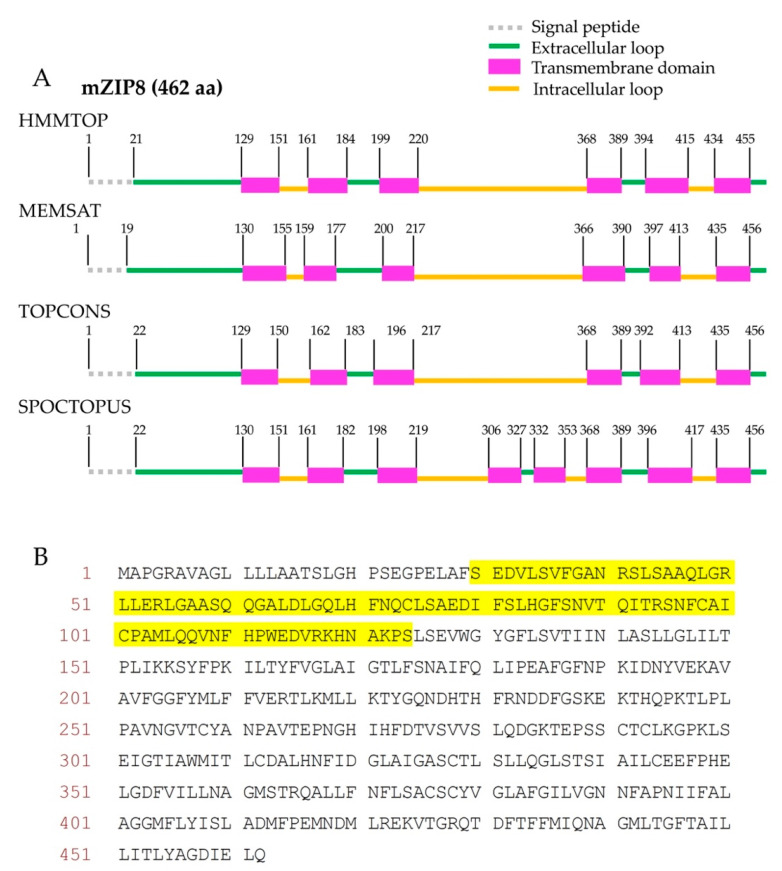
Graphical representation of the topological prediction of mZIP8 by four computational programs. (**A**) The programs HMMTOP, MEMSAT, TOPCONS, and SPOCTOPUS were used to identify putative transmembrane domains and signal peptide in mZIP8 protein. The locations of soluble segments (extracellular or cytosolic loops) and the positions of predicted transmembrane helices are indicated. The number denotes the first amino acid of each segment. (Please note that the length of each segment was not proportional to the actual number of amino acids). (**B**) The amino acid (aa) sequences selected for mZIP8 antigen are highlighted in yellow.

The mZIP8 is a polytopic membrane protein. To select suitable sequences for the antigen production, the transmembrane regions and signal peptide sequences within a polytopic protein need to be avoided [27,28]. Therefore, to design the sequence for the antigen production, we first established the topology model for mZIP8 by using four computational programs HMMTOP, MEMSAT, TOPCONS, and SPOCTOPUS. These four programs have been previously reported to retain high levels of accuracy for topological prediction [29]. For mZIP8 protein, the programs HMMTOP, MEMSAT, and TOPCONS predicted six transmembrane domains with four extracellular and three cytoplasmic loops, whereas the program SPOCTOPUS predicted eight transmembrane helices with five extracellular and four cytoplasmic loops (Figure 1A). Furthermore, all four programs consistently predicted the presence of an extended extracellular amino terminus (N terminus) and a cleavable signal peptide sequence near the start of mZIP8’s amino acid sequence with only minor variations in the predicted cleavage sites (Figure 1A). We avoided the signal peptide sequence and transmembrane helices, and selected the N-terminal extracellular region of mZIP8 for the antigen production (Figure 1B).

### 3.2. Construction of the Plasmid Expression and Purification of the GST Fusion Protein as the Antigen

**Figure 2:**
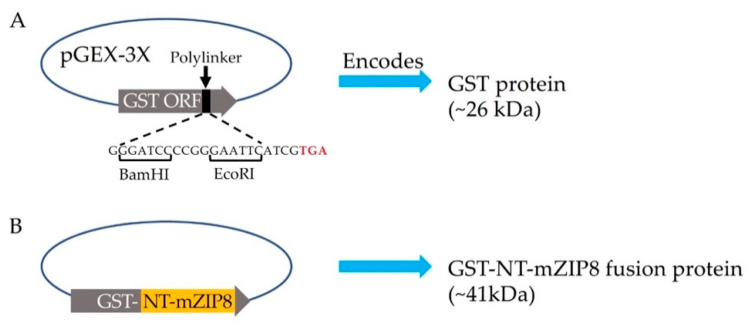
Schematic representation of pGEX-3X vector and the construction strategy for the plasmid encoding GST–NT-mZIP8. (**A**) The pGEX-3X vector contains the open reading frame (ORF) of GST followed by an expanded polylinker with restriction-enzyme cut sites upstream of the stop codon TAG (red letters). (**B**) the N-terminal sequences encoding serine 30 to serine 124 of mZIP8 (NT-mZIP8, orange) were cloned into pGEX-3X vector using BamHI/EcoRI sites to generate the recombinant plasmid pGEX-3X-GST–NT-mZIP8. The predicted molecular mass for GST was 26 kDa, and the anticipated product size for GST–NT-mZIP8 was 41 kDa.

**Figure 3:**
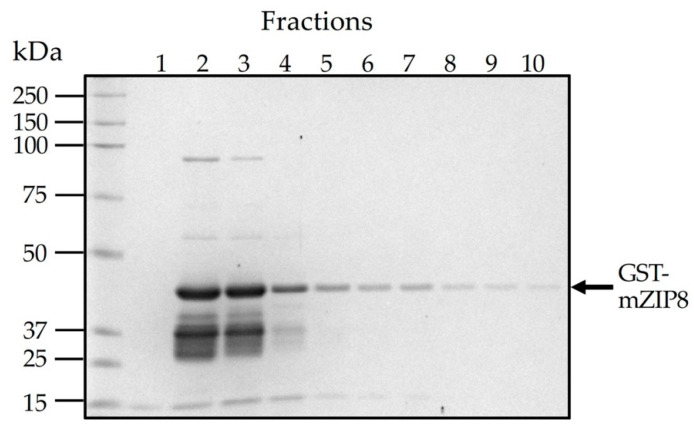
Purification of GST–NT-mZIP8 fusion protein from *E. Coli* transformed with the pGEX-3X recombinant vector. An overnight culture of *E. coli* cells transformed with the pGEX-3X-NT-mZIP8 vector was diluted in fresh LB media and grown for 1 h. After adding IPTG to the culture at a final concentration of 1 mM, the culture was grown for an additional 3 h. Cells were lysed, and the lysates were applied to the glutathione sepharose to allow the binding of GST fusion proteins. The elution buffer was added to elute the GST fusion proteins (10 consecutive fractions of the eluate were collected, 1 mL/fraction). SDS-PAGE gel loaded with different fractions of GST–mZIP8 was stained with Coomassie blue.

To facilitate the production and purification of antigen, the N-terminal sequence encoding serine 30 to serine 124 of mZIP8 (NT-mZIP8) was cloned into a pGEX-3X vector to allow the expression of the GST fusion protein. GST naturally occurs as a roughly 26 kDa protein (Figure 2A); the expected size of recombinant GST–NT-mZIP8 was predicted to be ~41 kDa (Figure 2B). After vector construction, the pGEX-3X plasmids encoding the GST fusion protein were transformed into *E. Coli*. The transformed bacteria were then cultured with 1 mM IPTG to induce the expression of GST fusion proteins. The lysates from IPTG-induced culture were assessed by SDS-PAGE, and the lysate without induction was used as the control to confirm the successful induction of GST fusion protein. The GST–NT-mZIP8 fusion protein was subsequently purified using immobilized glutathione sepharose column and analyzed by SDS-PAGE. Coomassie blue staining was applied to assess the identity of purified antigen. The band of the recombinant GST–NT-mZIP8 at the corresponding molecular size around 41 kDa could predominantly be observed in the SDS-PAGE gel (Figure 3), confirming the presence of desired antigen product.

### 3.3. Purification of Anti-mZIP8 Antibody

**Figure 4:**
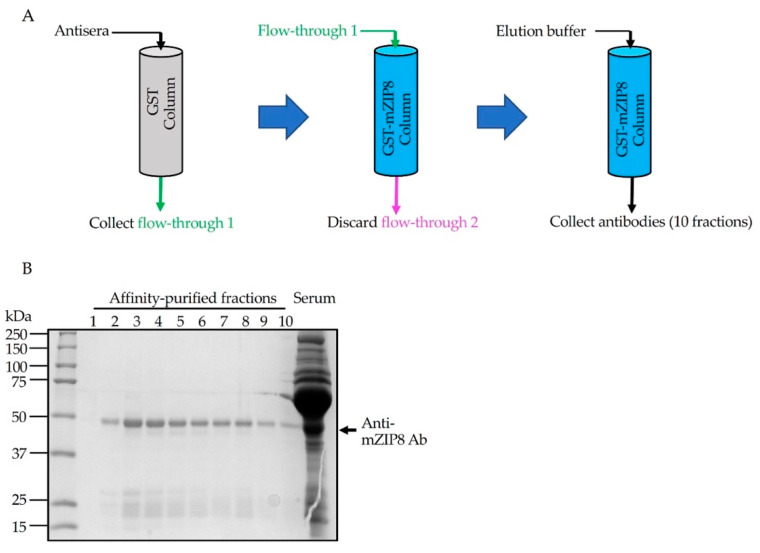
Purification of anti-mZIP8 antibody. (**A**) A schematic illustration of the affinity purification procedure. Rabbit antisera were diluted in binding buffer. The diluted sera were first applied to GST-linked sepharose column (GST column) three times to deplete anti-GST antibodies. The flow-through (flow-through 1) was then applied to the GST–NT-mZIP8-linked sepharose column (GST–mZIP8 column) to allow the binding of anti-mZIP8 antibody. The flow-through (flow-through 2) was discarded and the elution buffer was applied to elute antibodies (10 sequential fractions were collected, 1 mL/fraction). (**B**) Coomassie staining of SDS-PAGE gel loaded with different fractions of anti-mZIP8 antibodies, together with the original antiserum. The pooled antibodies from fractions 2, 3, and 4 were further used for antibody validation experiments.

To generate antigen-specific polyclonal antibody, rabbits were immunized with purified GST–NT-mZIP8 according to the standard procedure (Performed in the Pocono Rabbit Farm and Laboratory). Seventy days after the immunization, rabbit antisera were collected and affinity chromatography was used for the antibody purification [30]. Anti-GST antibodies were first depleted by affinity purification using GST-immobilized glutathione sepharose. The unbound flow-through from the GST column was collected and subsequently passed three times over the sepharose column-linked with GST–NT-mZIP8 to allow the binding of anti-mZIP8 antibodies (Figure 4A). The bound antibodies were eluted using antibody elution buffer. A total of ten sequential fractions from the final eluate, together with the initial antisera, were evaluated by Coomassie blue staining of SDS-PAGE gels performed under reducing conditions. The anti-mZIP8 antibody appeared abundantly at an apparent molecular mass of ~50 kDa, corresponding to the heavy chain of IgG, and the lower ~25 kDa bands could represent the IgG light chains (Figure 4B). Fractions 2, 3, and 4 of the final eluate appeared to contain higher amounts of antibodies, and were used for further antibody validation experiments.

### 3.4. Validation of Antibodies in Cells with Ectopic Overexpression of mZIP8

**Figure 5:**
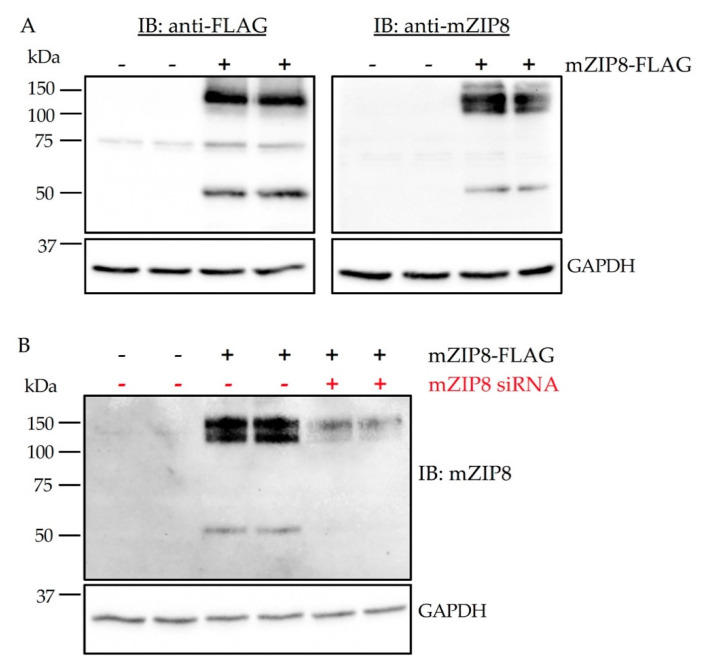
Validation of the anti-mZIP8 antibody in HEK293 cells. (**A**) Affinity-purified anti-mZIP8 antibody was validated in HEK293 cells overexpressing FLAG-tagged mZIP8 (+). Fourty-eight hours after transfection with plasmid encoding mZIP8, HEK293 cells were lysed and analyzed by immunoblotting (IB) with anti-FLAG or anti-mZIP8 antibodies. (**B**) HEK293 cells were transfected with the control vector (-) or a vector expressing FLAG-tagged mZIP8 (+). Twenty-four hours later, cells were transfected with negative control siRNA (-) or ZIP8-targeting siRNA (+). siRNA-mediated knockdown confirmed the identity of mZIP8 protein. The siRNA transfection was carried out for 24 h prior to immunoblotting analysis. GAPDH was used as the loading control.

Proper antibody validation for the immunoblotting assay is to demonstrate that an antibody can detect its target protein when bound to immobilization membranes [31]. To evaluate the specificity of anti-mZIP8 antibody, we first performed immunoblotting analysis of HEK293 cells overexpressing FLAG-tagged mZIP8. The anti-mZIP8 antibody detected primarily two bands, similar to the band pattern detected by the anti-FLAG antibody (Figure 5A). The 50 kDa band represents the predicted molecular mass of mZIP8 and the band at ~150 kDa may correspond to a multimer form of mZIP8.

To further determine the specificity of the mZIP8 antibody, we transiently transfected HEK293 cells with a plasmid encoding FLAG-tagged mZIP8. After plasmid transfection, cells were transfected with either negative control or mZIP8-targeting siRNA. Immunoblotting results indicate that siRNA transfected cells had a marked reduction in ZIP8 expression detected by the anti-mZIP8 antibody, verifying that the signals detected by the antibody indeed represent mZIP8 (Figure 5B).

### 3.5. The Anti-mZIP8 Antibody Can Recognize Endogenous Protein in Mouse Tissues

**Figure 6:**
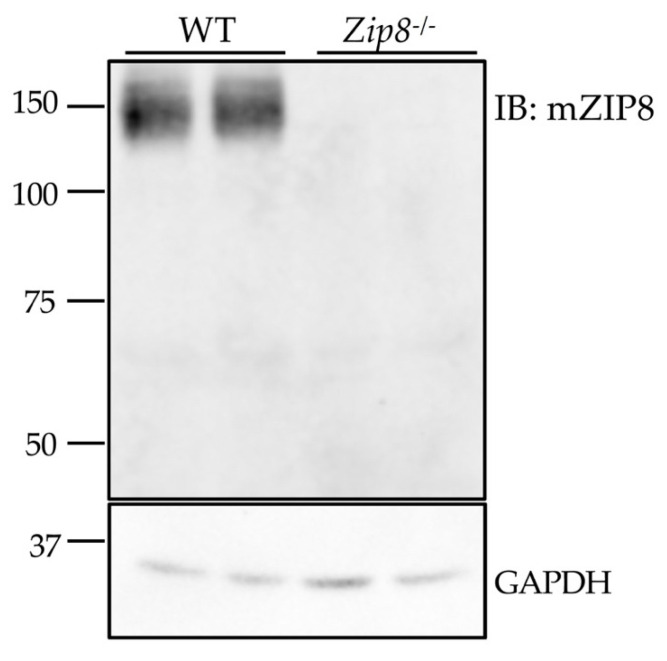
Validation of the anti-mZIP8 antibody using tissues from *Zip8* knockout mice. The anti-mZIP8 antibody was used in the immunoblotting (IB) assay to detect endogenous ZIP8 in the lungs of wild-type (WT) and *Zip8*^-/-^ mice. GAPDH was used as the loading control.

The use of cells that overexpress the antibody’s target protein provides useful information about the specificity of antibody, but the use of tissues from gene knockout animals serves as a fundamental approach to demonstrate whether the antibody can detect the endogenous protein [32]. Due to the lack of suitable antibodies, previous immunoblotting analyses that convincingly demonstrate endogenous ZIP8 protein expression in mouse tissues are limited. Therefore, we collected tissue samples from wild-type (WT) and *Zip*8^-/-^ mice to further carry out antibody validation. We found that the anti-mZIP8 antibody detects endogenous ZIP8 in the mouse lung at around 150 kDa, and that this immunoreactive band deceases in the tissue from *Zip8*^-/-^ mice, further verifying the specificity of anti-mZIP8 antibody (Figure 6). The detected band size of ~150 kDa is consistent with a previous report that in human lung epithelial A549 cells, the endogenous ZIP8 is detected only at ~150 kDa [33].

**Figure 7:**
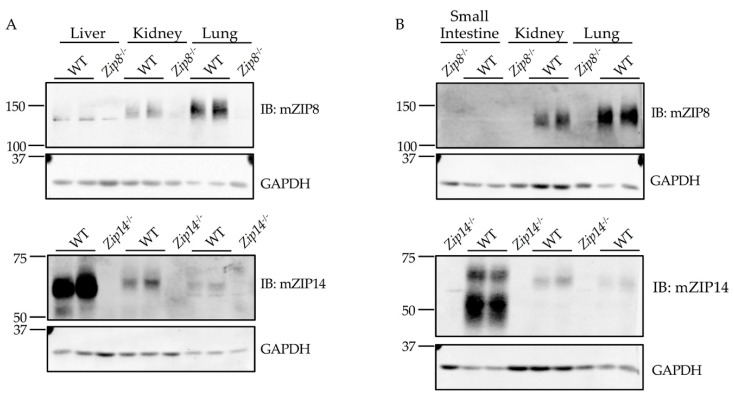
ZIP8 protein is enriched in the lung, but not in the liver or small intestine. The lung, kidney and (**A**) liver or (**B**) small intestine from wild-type (WT) and *Zip*8^-/-^ or *Zip*14^-/-^ mice were homogenized and analyzed by immunoblotting (IB). GAPDH was used as the loading control. The kidney samples were used in both (**A**,**B**) to serve as a tissue control because both ZIP8 and ZIP14 can be detected in the WT mouse kidney. Our results indicate that ZIP8 protein is highly expressed in the lung, and that ZIP14 is enriched in both the liver and small intestine.

The liver and small intestine are two major organs involved in regulating systemic manganese homeostasis [34]. Previous studies have determined that the mRNA level of ZIP8 is highest in the lung [5,35]. However, the protein levels of ZIP8 in the lung, liver, and small intestine have not been examined. The generation of anti-mZIP8 antibody allowed us to further compare ZIP8 protein expression in different mouse tissues using the immunoblotting assays. We revealed that, in contrast to ZIP14 which is highly expressed in the liver and small intestine, ZIP8 protein is highly enriched in the lung, but not in the liver or small intestine (Figure 7A,B). This finding may suggest a distinct role for ZIP8 in manganese metabolism of the lung.

Together, the anti-mZIP8 antibody generated in this study could be consistently applied in the immunoblotting assays to detect the endogenous mZIP8 protein, and would serve as a valuable tool for further studies involving the detection of mZIP8 protein at tissue levels.

## 4. Discussion

Antibodies are among the most frequently used reagents in biological research. In this study, we report on the production and validation of an antibody against the mouse metal transporter ZIP8. ZIP8 is a polytopic membrane protein. Studies using in vitro cell cultures and epitope-tagged ZIP8 variants have revealed that ZIP8 can mediate the influx of manganese into cells [4,6,36]. In humans, mutations in *ZIP8* lead to severe manganese deficiency [37,38,39], indicating that the primary function of ZIP8 is to regulate body manganese metabolism. However, the precise process and mechanisms of this regulation have not been fully determined.

Mouse models are widely used to provide insights into the mechanisms underlying human disorders [40]. Recently, it has been reported that the *Zip8*^-/-^ mice recapitulate the key symptoms of patients with *ZIP8* mutations [41], and therefore could serve as an invaluable tool for the understanding of mechanisms underlying human disorders associated with the loss of ZIP8. However, the use of mouse models to further investigate the function and regulation of ZIP8 was hindered by lack of reliable antibodies that can consistently detect endogenous mZIP8 protein.

To generate an antibody for mZIP8, we performed bioinformatic analyses to determine a base amino acid sequence for the design of an antigen. After identifying the potential signal peptide cleavage site, the N-terminal extracellular region of mZIP8 covering about 100 amino acids was selected and cloned into the pGEX-3X vector to produce a GST fusion protein for immunization. This strategy was taken for two reasons: first, the terminal region sequences are more immunogenic compared to core peptides [10,11]; second, because of the relatively larger sizes, GST fusion proteins typically contain more potentially immunogenic epitopes than the synthetic short peptides. Using this approach, we successfully generated a polyclonal anti-mZIP8 antibody. We also provided convincing results to demonstrate that this antibody can be reliably applied in immunoblotting assays to detect both mZIP8 ectopically expressed in cells and endogenous mZIP8 protein in mouse tissues. Therefore, this newly generated antibody will serve as a valuable tool to study the function and regulation of ZIP8 using immunoblotting assays. Future studies are needed to evaluate whether this antibody could be used in other antibody-based assays, such as immunohistochemistry and immunoprecipitation analyses.

From a practical standpoint, this strategy of generating an anti-mZIP8 antibody was based on a combination of bioinformatic analyses and the production of a GST fusion protein [20,42,43]. This approach can be directly used by other general laboratories. A similar approach was used to generate an anti-human ZIP8 antibody by Zang et al. [44]. Since antibodies are critical to many types of research, our results may offer valuable insights into the future development of antibodies against polytopic membrane proteins, especially for those with extended terminal domains.

## References

[1] Aschner M., Guilarte T.R., Schneider J.S., Zheng W. (2007). Manganese: Recent advances in understanding its transport and neurotoxicity. Toxicol. Appl. Pharmacol..

[2] Guilarte T.R. (2010). Manganese and Parkinson’s disease: A critical review and new findings. Environ. Health Perspect..

[3] Horning K.J., Caito S.W., Tipps K.G., Bowman A.B., Aschner M. (2015). Manganese Is Essential for Neuronal Health. Annu. Rev. Nutr..

[4] He L., Girijashanker K., Dalton T.P., Reed J., Li H., Soleimani M., Nebert D.W. (2006). ZIP8, member of the solute-carrier-39 (SLC39) metal-transporter family: Characterization of transporter properties. Mol. Pharmacol..

[5] Wang C.Y., Jenkitkasemwong S., Duarte S., Sparkman B.K., Shawki A., Mackenzie B., Knutson M.D. (2012). ZIP8 is an iron and zinc transporter whose cell-surface expression is up-regulated by cellular iron loading. J. Biol. Chem..

[6] Nebert D.W., Galvez-Peralta M., Hay E.B., Li H., Johansson E., Yin C., Wang B., He L., Soleimani M. (2012). ZIP14 and ZIP8 zinc/bicarbonate symporters in Xenopus oocytes: Characterization of metal uptake and inhibition. Metallomics.

[7] Choi E.K., Nguyen T.T., Gupta N., Iwase S., Seo Y.A. (2018). Functional analysis of SLC39A8 mutations and their implications for manganese deficiency and mitochondrial disorders. Sci. Rep..

[8] Trier N.H., Hansen P.R., Houen G. (2012). Production and characterization of peptide antibodies. Methods.

[9] Trier N., Hansen P., Houen G. (2019). Peptides, Antibodies, Peptide Antibodies and More. Int. J. Mol. Sci..

[10] Lee B.S., Huang J.S., Jayathilaka L.P., Lee J., Gupta S. (2016). Antibody Production with Synthetic Peptides. Methods Mol. Biol..

[11] Liang T.C., Luo W., Hsieh J.T., Lin S.H. (1996). Antibody binding to a peptide but not the whole protein by recognition of the C-terminal carboxy group. Arch. Biochem. Biophys..

[12] Brown M.C., Joaquim T.R., Chambers R., Onisk D.V., Yin F., Moriango J.M., Xu Y., Fancy D.A., Crowgey E.L., He Y. (2011). Impact of immunization technology and assay application on antibody performance--a systematic comparative evaluation. PLoS ONE.

[13] McCusker E.C., Bane S.E., O’Malley M.A., Robinson A.S. (2007). Heterologous GPCR expression: A bottleneck to obtaining crystal structures. Biotechnol. Prog..

[14] Venkatakrishnan A.J., Deupi X., Lebon G., Tate C.G., Schertler G.F., Babu M.M. (2013). Molecular signatures of G-protein-coupled receptors. Nature.

[15] Bill R.M., Henderson P.J., Iwata S., Kunji E.R., Michel H., Neutze R., Newstead S., Poolman B., Tate C.G., Vogel H. (2011). Overcoming barriers to membrane protein structure determination. Nat. Biotechnol..

[16] Sarkar C.A., Dodevski I., Kenig M., Dudli S., Mohr A., Hermans E., Pluckthun A. (2008). Directed evolution of a G protein-coupled receptor for expression, stability, and binding selectivity. Proc. Natl. Acad Sci. USA.

[17] Freigassner M., Pichler H., Glieder A. (2009). Tuning microbial hosts for membrane protein production. Microb. Cell Fact..

[18] Huang R., Kiss M.M., Batonick M., Weiner M.P., Kay B.K. (2016). Generating Recombinant Antibodies to Membrane Proteins through Phage Display. Antibodies (Basel).

[19] Forsstrom B., Axnas B.B., Rockberg J., Danielsson H., Bohlin A., Uhlen M. (2015). Dissecting antibodies with regards to linear and conformational epitopes. PLoS ONE.

[20] Nilsson P., Paavilainen L., Larsson K., Odling J., Sundberg M., Andersson A.C., Kampf C., Persson A., Al-Khalili Szigyarto C., Ottosson J. (2005). Towards a human proteome atlas: High-throughput generation of mono-specific antibodies for tissue profiling. Proteomics.

[21] Uhlen M., Oksvold P., Fagerberg L., Lundberg E., Jonasson K., Forsberg M., Zwahlen M., Kampf C., Wester K., Hober S. (2010). Towards a knowledge-based Human Protein Atlas. Nat. Biotechnol..

[22] Tusnady G.E., Simon I. (2001). The HMMTOP transmembrane topology prediction server. Bioinformatics.

[23] Jones D.T., Taylor W.R., Thornton J.M. (1994). A model recognition approach to the prediction of all-helical membrane protein structure and topology. Biochemistry.

[24] Tsirigos K.D., Peters C., Shu N., Kall L., Elofsson A. (2015). The TOPCONS web server for consensus prediction of membrane protein topology and signal peptides. Nucleic Acids Res..

[25] Viklund H., Bernsel A., Skwark M., Elofsson A. (2008). SPOCTOPUS: A combined predictor of signal peptides and membrane protein topology. Bioinformatics.

[26] Wang B., He L., Dong H., Dalton T.P., Nebert D.W. (2011). Generation of a Slc39a8 hypomorph mouse: Markedly decreased ZIP8 Zn^2+^/(HCO_3_^−^)_2_ transporter expression. Biochem. Biophys. Res. Commun..

[27] Uhlen M., Bjorling E., Agaton C., Szigyarto C.A., Amini B., Andersen E., Andersson A.C., Angelidou P., Asplund A., Asplund C. (2005). A human protein atlas for normal and cancer tissues based on antibody proteomics. Mol. Cell Proteomics.

[28] Lindskog M., Rockberg J., Uhlen M., Sterky F. (2005). Selection of protein epitopes for antibody production. Biotechniques.

[29] Reddy A., Cho J., Ling S., Reddy V., Shlykov M., Saier M.H. (2014). Reliability of nine programs of topological predictions and their application to integral membrane channel and carrier proteins. J. Mol. Microbiol. Biotechnol..

[30] Frangioni J.V., Neel B.G. (1993). Solubilization and purification of enzymatically active glutathione S-transferase (pGEX) fusion proteins. Anal. Biochem..

[31] Pillai-Kastoori L., Heaton S., Shiflett S.D., Roberts A.C., Solache A., Schutz-Geschwender A.R. (2020). Antibody validation for Western blot: By the user, for the user. J Biol Chem.

[32] Bordeaux J., Welsh A., Agarwal S., Killiam E., Baquero M., Hanna J., Anagnostou V., Rimm D. (2010). Antibody validation. Biotechniques.

[33] Scheiber I.F., Alarcon N.O., Zhao N. (2019). Manganese Uptake by A549 Cells is Mediated by Both ZIP8 and ZIP14. Nutrients.

[34] Winslow J.W.W., Limesand K.H., Zhao N. (2020). The Functions of ZIP8, ZIP14, and ZnT10 in the Regulation of Systemic Manganese Homeostasis. Int. J. Mol. Sci..

[35] Yue F., Cheng Y., Breschi A., Vierstra J., Wu W., Ryba T., Sandstrom R., Ma Z., Davis C., Pope B.D. (2014). A comparative encyclopedia of DNA elements in the mouse genome. Nature.

[36] Fujishiro H., Yano Y., Takada Y., Tanihara M., Himeno S. (2012). Roles of ZIP8, ZIP14, and DMT1 in transport of cadmium and manganese in mouse kidney proximal tubule cells. Metallomics.

[37] Park J.H., Hogrebe M., Gruneberg M., DuChesne I., von der Heiden A.L., Reunert J., Schlingmann K.P., Boycott K.M., Beaulieu C.L., Mhanni A.A. (2015). SLC39A8 Deficiency: A Disorder of Manganese Transport and Glycosylation. Am. J. Hum. Genet..

[38] Boycott K.M., Beaulieu C.L., Kernohan K.D., Gebril O.H., Mhanni A., Chudley A.E., Redl D., Qin W., Hampson S., Kury S. (2015). Autosomal-Recessive Intellectual Disability with Cerebellar Atrophy Syndrome Caused by Mutation of the Manganese and Zinc Transporter Gene SLC39A8. Am. J. Hum. Genet..

[39] Riley L.G., Cowley M.J., Gayevskiy V., Roscioli T., Thorburn D.R., Prelog K., Bahlo M., Sue C.M., Balasubramaniam S., Christodoulou J. (2017). A SLC39A8 variant causes manganese deficiency, and glycosylation and mitochondrial disorders. J. Inherit. Metab. Dis..

[40] Rosenthal N., Brown S. (2007). The mouse ascending: Perspectives for human-disease models. Nat. Cell Biol..

[41] Lin W., Vann D.R., Doulias P.T., Wang T., Landesberg G., Li X., Ricciotti E., Scalia R., He M., Hand N.J. (2017). Hepatic metal ion transporter ZIP8 regulates manganese homeostasis and manganese-dependent enzyme activity. J. Clin. Investig..

[42] Berglund L., Bjorling E., Jonasson K., Rockberg J., Fagerberg L., Al-Khalili Szigyarto C., Sivertsson A., Uhlen M. (2008). A whole-genome bioinformatics approach to selection of antigens for systematic antibody generation. Proteomics.

[43] Larsson M., Graslund S., Yuan L., Brundell E., Uhlen M., Hoog C., Stahl S. (2000). High-throughput protein expression of cDNA products as a tool in functional genomics. J. Biotechnol..

[44] Zang Z., Xu Y., Lau A.T. (2015). Preparation of highly specific polyclonal antibody for human zinc transporter ZIP8. Acta. Biochim. Biophys. Sin. (Shanghai).

